# A Novel Treatment of Postpartum Depression and Review of Literature

**DOI:** 10.7759/cureus.22373

**Published:** 2022-02-18

**Authors:** Jennifer Yoon, Jason Gu, Katherine B Martin

**Affiliations:** 1 Psychiatry, University of South Florida Morsani College of Medicine, Tampa, USA; 2 Psychiatry, Lehigh Valley Health Network, Allentown, USA

**Keywords:** post partum depression, post-partum mood disorder, perinatal psychiatry, peripartum depression, perinatal mental health

## Abstract

Early-onset postpartum depression has been shown to have a unique neurobiological basis compared to major depressive disorder, implying a need for targeted treatments such as the recent Food and Drug Administration (FDA)-approved brexanolone. In this case report, a woman with a past medical history of major depressive disorder was diagnosed with postpartum depression due to worsening mood with suicidal and homicidal ideations. She was treated with vilazodone and aripiprazole with good effect after consideration of her past medication trials. Her regimen is unique in clinical practice and not reported in current literature for the treatment of postpartum depression. It may represent a safe and effective medication choice, especially in the context of current first-line treatments that have a high treatment failure rate. More research is needed to find treatments that address the unique challenges of postpartum women.

## Introduction

Postpartum depression (PPD) is the most common complication of pregnancy [1]. Studies have shown that it can have significant long-term effects on both the mother and her newborn [2]. It is associated with negative maternal caretaking behaviors as well as inferior language skills and IQ development in the child [2]. While early treatment of PPD is key for reducing these outcomes, there are unique challenges in treating this population. This report will review a recent case of PPD in which a woman presented with suicidal and homicidal ideation one month after delivery. Using the case as a catalyst, this paper will review updated pharmacotherapy treatment options for PPD.

## Case presentation

The patient was a 30-year-old female with a history of major depressive disorder (MDD), self-injurious behaviors (cutting as recently as seven years ago), and attention deficit hyperactivity disorder (ADHD). More recently she had been suffering from PPD after the birth of her child one month earlier. She presented to the emergency department with two days of worsening depression along with suicidal and homicidal ideation. Her plan for suicide was to overdose on medications or stab herself with a knife. Her homicidal thoughts were less specific but were directed toward her husband and child. She also endorsed poor sleep, energy, and concentration. She denied any recent substance use and was not breastfeeding. She was overwhelmed by having to care for her newborn, perform household chores, and take care of her twenty pets. Prior to her most recent pregnancy, she was treated with vilazodone 40mg daily and aripiprazole 5mg daily with a good effect on her mood. She had stopped these medications during her pregnancy, but her mood had managed to remain stable until after her delivery. 

On account of her symptomatology, she was admitted voluntarily to the inpatient psychiatric unit. She was diagnosed with MDD, recurrent, severe, without psychotic features. She was restarted back on vilazodone 40mg daily and aripiprazole 5mg daily. A family meeting was held to bolster her social support at home. After her third day of admission, her suicidal and homicidal ideation had resolved, and she was able to be discharged home with continued medication management as well as a referral for outpatient therapy.

## Discussion

The postpartum period is a unique time as there are significant fluctuations in a woman’s hormone levels that are associated with the pathophysiology of PPD [3]. Furthermore, there are critical psychosocial stressors that a new mother faces in adjusting to her new role. For many women, there is a stigma in seeking pharmacological treatment of PPD [4]. As in this case, the decision of whether to continue psychiatric medication during the pregnancy and postpartum periods can also be a stressor. Furthermore, as also was seen in this case, PPD can present as a clinical emergency as it can lead to suicidal ideation, a suicide attempt, or more rarely, infanticide [5,6]. Therefore, all mental health professionals must be familiar and comfortable with diagnosing and treating PPD.

Current pharmacotherapy treatment options of PPD

Postpartum depression is often clinically treated as a subtype of MDD. Consequently, selective serotonin reuptake inhibitors (SSRIs) are the most popular choice of pharmacotherapy [7]. Furthermore, SSRIs generally have a good safety profile for breastfeeding mothers. Paroxetine and sertraline are least likely to be detected in infant plasma [7]. Current evidence on the effectiveness of SSRIs for PPD shows that pharmacotherapy alone can be as effective as cognitive-behavioural therapy (CBT) with pharmacotherapy [7]. However, these studies have been criticized for lack of long-term follow-up and underpowered sample sizes. Additionally, the use of SSRIs is associated with a remission rate of only up to 60%, which demonstrates the need for more diverse and effective treatments for PPD [8].

The Diagnostic and Statistical Manual of Mental Disorders, Fifth Edition (DSM-5), labels PPD as “MDD with peripartum onset,” which is defined as the onset of mood symptoms during pregnancy or within four weeks after delivery. However, PPD has also been variably defined as depression that occurs at three months, six months, or 12 months after delivery [9]. Recent studies have found that the onset of PPD within the first eight weeks of childbirth is distinct from MDD whereas late-onset PPD is more like MDD outside of the postpartum period [8,10]. This finding is due to fluctuating levels of reproductive hormones that occur soon after birth. Therefore, there is likely a unique etiology for PPD as compared to MDD [10].

Drawing on this unique etiology of PPD, brexanolone, an allopregnanolone analog, became the first drug to be approved by the Food and Drug Administration (FDA) specifically for PPD in 2019 [11]. Its use is based on research that found allopregnanolone to be a neuroactive hormone that sharply decreases after birth. This decrease leads to overactivity of gamma-aminobutyric acid (GABA) receptor activation and triggers PPD [12]. Clinical trials of brexanolone have shown a significant reduction in symptoms of moderate to severe PPD. These results are promising as this group of patients had often been resistant to SSRIs. While it is detected in breastmilk, infant exposure is very low. However, its use is currently restricted to certified healthcare facilities and must be infused over 60 hours. These factors, along with its cost of approximately $39,000 for the drug and its administration, have significantly limited its access [13].

There are other emerging treatments for PPD that show promise. Transcranial magnetic stimulation has been studied in patients that have PPD with significant effects in both open and double-blinded studies [14]. Omega-3 fatty acid supplements have been studied for both prevention and treatment of PPD, due to evidence that links PPD with reduced fatty acids [8]. 

Use of vilazodone and aripiprazole in PPD

In our case, the patient was treated with a combination of vilazodone and aripiprazole because these are the medications she did well on before pregnancy. This strategy is often employed when choosing a medication for any patient with depression [15]. With specific regard to PPD, the decision is also based on the balance of the risks and benefits of the proposed treatment [16]. The patient in this case was not breastfeeding. Therefore, the risks of restarting her medications were quite low and the benefits quite clear. However, it is more often the case that physicians are treating PPD in patients without a psychiatric history, leading to recommendations that may not have clear clinical significance.

Had our patient been pregnant or breastfeeding, it would have been more difficult to counsel her on the potential risks of vilazodone due to a lack of data. There are no human trials on vilazodone in this population, but animal trials have shown adverse effects in the fetus [17]. A case study of a woman who unexpectedly became pregnant while on vilazodone 40mg daily resulted in a normal birth without complication [18]. In our case presentation, the patient's extensive psychiatric history and the acuity of her presenting symptoms were factors in restarting her on vilazodone rather than choosing a “safer” choice such as an SSRI. Furthermore, there has been no research comparing the efficacy of vilazodone to SSRIs for PPD. Therefore, the question remains whether vilazodone could be a more effective treatment.

Like vilazodone, little data is also available on aripiprazole in pregnancy or breastfeeding. Until recently, human data for aripiprazole was limited to case reports. More recently, two larger prospective studies were relatively reassuring and did not indicate a higher risk from treatment with aripiprazole as compared to other second-generation antipsychotics during pregnancy, postpartum, and lactation periods [19]. In breastfeeding, insufficient milk from reduced prolactin release had been a concern, but no adverse reactions have been seen [19].

## Conclusions

Postpartum depression can have long-term negative effects on a mother and her child. Treatment regimens can be challenging due to the changes in mood and the physiology of a woman adjusting to her new role as a mother. There is a change in paradigm in considering PPD as a separate entity from MDD and this change may lead the way to novel pharmacotherapy strategies. Currently, decisions for treatment are made by weighing benefits and risks which can be challenging when there is a lack of reliable data. Continued research is essential to improving treatment strategies for this population.
